# Long-Term Associations of Early-Life Human Milk Oligosaccharide Intake with Allergic Disease Development and Gut Microbiota Profiles in 5-Year-Old Children

**DOI:** 10.3390/nu18040624

**Published:** 2026-02-13

**Authors:** Ruixin Kou, Che Pan, Xiaolong Xing, Jin Wang, Sinéad T. Morrin, Rachael H. Buck, Xiang Li, Yingyi Mao, Shuo Wang

**Affiliations:** 1 Key Laboratory of Special Diet Nutrition and Health Research of China National Light Industry Council, School of Medicine, Nankai University, Tianjin 300071, China; kourx@mail.nankai.edu.cn (R.K.); xingxl_pumc@163.com (X.X.); wangjin@nankai.edu.cn (J.W.); 2 Research Institute of Public Health, Nankai University, Tianjin 300071, China; 3 Abbott Nutrition Research & Development Center, Shanghai 200233, China; che.pan@abbott.com; 4 Abbott Nutrition Research & Development Center, Columbus, OH 43215, USA; sinead.morrin@abbott.com (S.T.M.); rachaelbuck64@gmail.com (R.H.B.); 5 Abbott Nutrition Research & Development Center, Singapore 138668, Singapore; xiang.li@abbott.com

**Keywords:** human milk oligosaccharides, allergic disease, gut microbiota, short-chain fatty acids, *Bifidobacterium adolescentis*

## Abstract

**Background**: Based on our extensive cohort study, the Maternal Nutrition and Infant Investigation (MUAI), this research investigated the associations between human milk oligosaccharide (HMO) intake during the postnatal period and allergic disease development and gut microbiome composition in early childhood through long-term follow-up. **Methods**: Human breast milk (HBM) samples at five lactation stages and fecal samples of infants and young children were collected. Children aged 5 years included in this study were categorized into allergic and non-allergic groups via standardized allergen testing. **Results**: The findings indicated that higher HMO intake levels across five distinct lactation periods may be linked to a reduced incidence of allergies in children. The consumption of six major structurally representative HMOs was significantly associated with alterations in the gut microbiota profiles of young children. Moreover, there were notable differences in gut microbiota composition between allergic and non-allergic children. Specifically, beneficial bacteria such as *Bifidobacterium*, *Akkermansia*, and *Ruminococcus* were significantly enriched, in addition to the levels of metabolite propionic acid, a beneficial short-chain fatty acid, which were notably higher in the non-allergic group. To further validate the relationship between *Bifidobacterium* abundance and early HMO intake, the analysis revealed that a differential strain biomarker, *Bifidobacterium adolescentis* (*B. adolescentis*), exhibited significant correlations with specific HMOs at different lactation stages, particularly showing a strong positive correlation with 2′-fucosyllactose (2′-FL) content. **Conclusions**: These findings suggest that early-life HMO intake is associated with long-term differences in allergic outcomes, potentially through modulation of gut microbiota composition, particularly the enrichment of *B. adolescentis*.

## 1. Introduction

Human breast milk (HBM) is widely regarded as the gold standard for early-life nutrition, owing to its rich composition of bioactive components and essential nutrients that fully support the nutritional needs of infants during the first months of life [1]. Breastfeeding is associated with immune maturation and developmental support of the gut microbiome in healthy infants and even later in life [2]. In recent years, crucial and distinctive potential bioactivities of human milk oligosaccharides (HMOs), the third most abundant solid constituent in HBM following lactose and lipids, have attracted extensive attention in studies investigating the benefits of breastfeeding. Particularly regarding early immune system programming, pre-clinical research has shown HMOs actively facilitate the development and functional maturation of mucosal, innate, and adaptive immunity [3,4]. More than 200 oligosaccharide structures have been identified in HBM by chromatography and mass spectrometry and are classified as fucosylated, neutral non-fucosylated (acetylated), and sialylated HMOs based on core structural modifications [5,6]. Characterization of the composition of HMOs is influenced by multiple factors, including maternal phenotypes regulated by the fucosyltransferase FUT2 [Secretion (Se) gene] and FUT3 [Lewis (Le) gene] genes. The main HMOs present in the HBM of mothers classified as secretory (Se+) are 2′-fucosyllactose (2′-FL), difucosyllactose (DFL), and lactose-*N*-fucosidase I (LNFP I), whereas non-secretory (Se-) mothers predominantly biosynthesize 3-FL and LNFP II [7,8]. Given the reported prebiotic benefits, several HMOs, including 2′-FL, 3-FL, 3′-Sialyllactose (3′-SL), 6′-SL, lacto-N-tetraose (LNT), and lacto-N-neo-tetraose (LNnT), have been approved as nutritional supplements in infants and young children foods [9].

Allergic diseases are a group of systemic and mucosal diseases including, but not limited to, food allergy (FA), allergic asthma (AAS), atopic dermatitis (AD), and eczema [10]. Allergic disease, listed by the World Health Organization (WHO) as one of the top three major diseases in the 21st century, remains highly prevalent globally, especially in infancy and early childhood [11,12]. A wide range of ingested/inhaled/contact allergens, including food (nuts, milk, eggs, etc.), inhalants (pollen, house dust, mites, etc.), and microorganisms (molds, bacteria, etc.), as well as insect toxins and medications (penicillin, sulfonamides, etc.), may induce an allergic reaction, which is defined as the body’s over-activation of an immune response to a harmless substance. Several studies suggest a possible association between breastfeeding and lower allergy prevalence in early childhood [13,14,15]. Moreover, both preclinical and clinical studies have consistently demonstrated that breastfed infants exhibit a significantly higher abundance of *Bifidobacterium* species in their gut microbiome compared to non-breastfed infants, with HMOs playing a central role as ‘bifidogenic factors’ [16,17]. Numerous studies have demonstrated that stable interactions between the gut microbiota and the immune system can produce complex, beneficial effects in the management of allergic diseases [18,19]. A recent study revealed that a deficiency in *Bifidobacteria*, and more specifically the absence of genes essential for HMO metabolism within the gut metagenome, is strongly associated with systemic inflammation and immune dysregulation during early life [20].

Within the initial weeks postpartum, feeding regimen supersedes birth mode as the predominant determinant of microbiota composition during this transitional developmental phase [4,21]. During breastfeeding, HMOs, which are not digested by human enzymes, are crucial for the initial *bifidobacteria*-dominated microbial colonization pattern of the distal colon in early life [22,23]. Shaping a balanced microbial environment may profoundly affect the onset and development of allergic diseases even later in life, and HMOs are reported to specifically proliferate commensals like *Bifidobacteria* and simultaneously resist infection due to pathogenic *Campylobacter jejuni*, *Escherichia coli*, and *Listeria monocytogenes* [24,25,26]. In addition to exerting prebiotic effects in the colon, low levels of HMOs are found to cross the enterocyte monolayer into the bloodstream to modulate systemic immunity [27,28]. It has been reported in both in vivo and in vitro studies that HMOs can maintain immune homeostasis by enhancing intestinal barrier function or by directly contacting the immune system, thereby alleviating allergic diseases [3,29]. Furthermore, HMO-supplemented infant formulas, particularly those using extensively hydrolyzed proteins or amino acid-based formulas, have been scientifically demonstrated to not only exhibit hypoallergenicity and be well-tolerated by infants with cow’s milk protein allergy (CMPA) but also partially correct gut microbial dysbiosis in this susceptible population through microbiota-directed mechanisms [30,31]. Nevertheless, the reported benefits of HMOs are not uniform across populations, as HMO composition and downstream microbiota responses are shaped by maternal genetics, diet, and environmental factors. In particular, maternal secretor status may significantly influence infant HMO exposure and subsequent microbiota and immune development, representing a potential confounding factor in observational analyses. In addition, most available human evidence is derived from short-term or early-infancy studies, highlighting the need for longitudinal investigations with clinically assessed outcomes.

Despite growing recognition of the bioactive potential of HMOs, critical gaps remain in long-term longitudinal studies examining how quantified HMO intake during defined lactation stages relates to allergic outcomes beyond infancy. While previous studies have provided important insights into early microbiota composition and immune development, many were cross-sectional, relied on surrogate markers, or had limited follow-up durations. Consequently, it remains unclear which specific gut microbial taxa may persistently mediate the association between early-life HMO exposure and subsequent allergy risk, highlighting the necessity and value of longitudinal studies, such as ours, that integrate quantitative HMO intake, long-term gut microbiome development, and clinically assessed allergy outcomes. Importantly, the relationships among HMO intake, gut microbiome development, and allergic outcomes are inherently complex and influenced by multiple interrelated factors, including genetic predisposition, environmental exposures, and maternal health-related behaviors, such that observational studies primarily reveal associations rather than causality.

Therefore, our study aimed to characterize HMO profiles across five lactation stages (0–400 days) in healthy breastfed mother–infant pairs and to longitudinally track gut microbiome development and allergic outcomes in children up to 5 years of age, with the primary objectives of examining associations between early-life HMO intake and allergy incidence and identifying bacterial taxa potentially mediating these microbiota–host interactions. Our observational study not only provides new insights into the association between the intake of HMOs early in life and allergy development in young children but also offers a theoretical basis for future mechanistic studies and for evaluating the potential benefits of HMO supplementation.

## 2. Materials and Methods

### 2.1. Study Design and Participants

**Figure 1:**
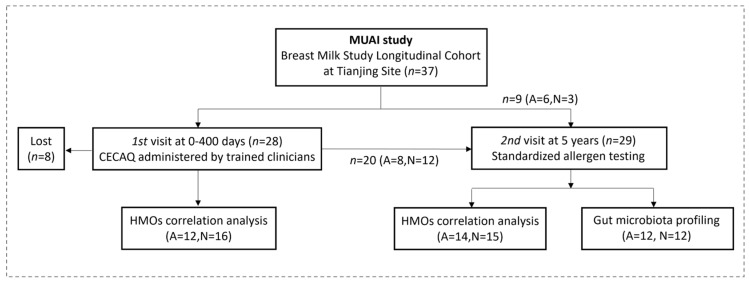
Flow chart of the study design and participant inclusion. This study was conducted as part of the Maternal Nutrition and Infant Investigation (MUAI) cohort. Mother–infant pairs were enrolled during infancy according to predefined recruitment and exclusion criteria. Allergic status during early life was assessed using the Comprehensive Early Childhood Allergy Questionnaire (CECAQ). And at the 5-year follow-up, children underwent standardized allergen testing for classification into allergic (A) and non-allergic (N) groups. MUAI, Maternal Nutrition and Infant Investigation; CECAQ, Comprehensive Early Childhood Allergy Questionnaire; HMO, human milk oligosaccharide; A, allergic; N, non-allergic.

This study was a part of the Maternal Nutrition and Infant Investigation (MUAI) study [32], aiming to investigate the effects of HMO intake in early life on the occurrence and development of allergic diseases in infants and young children. The study design is shown in Figure 1. At the 1st visit, from 10 November 2017 to 7 December 2018, the study population was recruited from antenatal clinics and classes at Tianjin Hospital of ITCWM Nankai Hospital, Tianjin, China. The recruitment and exclusion criteria for mother–infant pairs were consistent with our previously published study [33]: healthy pregnant women who had lived in Tianjin for more than two years, 20–35 years old, who planned to breastfeed for more than 3 months and had singleton pregnancies with an infant gestational age of 37–42 weeks and an Apgar score over 8. Subjects were excluded if they had any medical conditions during pregnancy or lactation. HBM samples were collected to detect the content of HMOs at five lactation stages: the colostrum period (0–5 days postpartum, CM), transitional milk period (10–15 days postpartum, TM), mature milk period (40–45 days postpartum, MM), the period during complementary food introduction (200–240 days postpartum, M2) and one year after delivery (300–400 days postpartum, M3). The milk samples were collected following rigorous protocols, as described in our previous work [33]. Three parallel measurements were performed for each sample during HMO analysis to guarantee precision. These five stages were selected as key time points for understanding HMO intake and its potential role in allergy development, balancing the need for accurate data and minimal participant discomfort. The Comprehensive Early Childhood Allergy Questionnaire (CECAQ)—a validated instrument specifically developed to assess allergic conditions, including food allergy, atopic dermatitis, and asthma, in infants and young children aged 1–5 years—was employed in this study [34].

At the 2nd visit in December 2023, 8 mother–child pairs were lost to follow-up due to participant withdrawal during the long-term follow-up period. To maximize the sample size while maintaining consistency with the study objectives, an additional 9 mother–child pairs with concerns about allergic conditions who continuously resided in Tianjin and had complete early-life HMO data were recruited from the same MUAI cohort. All included participants met the same predefined inclusion and exclusion criteria. Baseline maternal and infant characteristics of retained, lost-to-follow-up, and supplemented participants are summarized in Supplementary Table S1. At 5 years of age, children underwent fecal sample collection, dietary questionnaire assessment, CECAQ administration, and standardized clinical allergen testing. Allergen detection was performed at Tianjin Children’s Hospital and included a panel of 34 common inhalant and food allergens, with test results interpreted and diagnosed by experienced clinicians. All the children included had lived in Tianjin since birth, did not have major chronic diseases or consume antibiotics and pharmaceutical agents that may affect gut microbes for a long period or within 45 days of study visits, and did not have respiratory infections, diarrhea, or other infectious diseases during the study period. Data were collected by uniformly trained hospital doctors and medical students.

This study was conducted following the Declaration of Helsinki, and registered in the China Clinical Trial Center (registration number: ChiCTR1800015387). The research protocol was approved by the Medical Ethics Committee of Nankai University (approval number: NKUIRB2024103). Written informed consent was obtained from all participants before data collection.

### 2.2. Gut Microbiota Profiling

Fresh fecal samples were collected by parents using sterile containers and immediately placed in insulated boxes with dry ice. Samples were transported to the laboratory within 4 h, aliquoted under sterile conditions, and stored at −80 °C until analysis. The median storage duration from collection to DNA extraction was one week. To prevent freeze–thaw damage, samples were aliquoted into multiple tubes upon arrival, and only single-use aliquots were thawed for each analysis. All samples were processed in a single batch to eliminate technical batch effects. All samples were handled following the same standardized protocol to minimize potential technical variation.

Microbial total genomic DNA from infants’ and children’s feces was extracted, purified, and identified as previously described [35]. Universal primers 341F (5′-CCTAYGGGRBGCASCAG-3′) and 806R (5′-GGACTACNNGGGTATCTAAT-3′) were used to amplify the V3–V4 hypervariable region of the bacterial 16S rRNA gene. Raw paired-end sequencing reads (PE150) were processed for quality control. Adapter sequences and low-quality bases were trimmed according to standard filtering criteria (Phred quality score < Q20), and reads containing ambiguous nucleotides or nucleotides shorter than 50 bp were discarded. Paired-end reads were merged, and chimeric sequences were identified and removed using USEARCH against the Gold reference database. Samples yielding fewer than 30,000 clean reads or exhibiting Good’s coverage values below 99% were excluded to ensure adequate sequencing depth. Taxonomic assignment was performed using the RDP Classifier against the SILVA 138 database at an 80% confidence threshold. High-quality reads were clustered into operational taxonomic units (OTUs) based on 97% sequence similarity, followed by taxonomic assignment using a curated reference database. To account for differences in sequencing depth, data were normalized prior to downstream diversity and differential abundance analyses. Bioinformatic analysis, including species taxonomic analysis, alpha diversity analysis, principal component analysis (PCoA), and linear discriminant analysis (LEfSe) were performed on the NovoMagic platform (Novogene Genomics Technology Co., Ltd., Beijing, China). Spearman correlation analysis was used to explore the correlation between intestinal microbiota and allergic parameters.

### 2.3. Gas Chromatography–Mass Spectrometry (GC-MS) Analysis of SCFAs in Feces

The standard curve was constructed with standard mixtures of 0.05 μg/mL, 0.1 μg/mL, 0.5 μg/mL, 1 μg/mL, 5 μg/mL, 10 μg/mL, 25 μg/mL, 50 μg/mL, 100 μg/mL, and 250 μg/mL concentration gradients prepared with ether (R^2^ > 0.998), fully covering the detected concentration range. A 100 mg aliquot of fecal samples was taken from −80 °C storage, thawed on ice (single freeze–thaw cycle), and mixed with 100 μL 15% phosphoric acid and 100 μL 50 μg/mL internal standard isocaproic acid solution. Samples were processed immediately after thawing to prevent SCFA degradation. A volume of 400 μL ether was added for extraction, followed by homogenization (1 min), centrifugation (12,000 rpm, 4 °C, 10 min), and GC-MS analysis of the supernatant. Each sample was measured in duplicate. Analytical quality control included (i) isocaproic acid as internal standard to monitor extraction efficiency; (ii) spiked recovery tests showing 85–115% recovery for all SCFAs; (iii) limits of detection (LODs) and quantification (LOQs) of 0.01–0.05 μg/mL and 0.03–0.15 μg/mL, respectively (S/N ≥ 3); (iv) intra-day precision (RSD < 10%) and inter-day precision (RSD < 15%); and (v) duplicate injections with CV < 15%. The content of SCFAs in each sample was calculated according to the standard curve. All samples were processed for SCFA analysis within 1 week of collection to minimize pre-analytical variability.

### 2.4. Statistical Analysis

All statistical analyses were performed using Prism 8.0.1 (GraphPad, San Diego, CA, USA). For data that were not normally distributed, comparisons of HMO concentrations, SCFA levels, and microbiota features were conducted using Kruskal–Wallis one-way ANOVA with all-pairwise post hoc comparisons. To control for the false discovery rate arising from multiple testing across microbial taxa, SCFA levels, and related variables, *p*-values were adjusted using the Benjamini–Hochberg (FDR) correction. Spearman correlation analyses were performed to assess associations between gut microbiota features and allergic parameters, with interpretation taking into account multiple comparisons.

## 3. Results

### 3.1. Correlation Between HMO Intake and the Incidence of Allergic Diseases in Infants

To determine whether HMO intake through breastfeeding is associated with differences in the incidence of allergic disease in infants, we compared the intake levels of 17 HMOs at five lactation stages between allergic and non-allergic infants (Supplementary Table S2). The infants were grouped based on maternal CECAQ responses of whether their children exhibited allergy symptoms from the M2 to M3 lactation period. Of the 28 mother–child pairs, 12 mothers reported that their babies had experienced AD, FA, or eczema symptoms, and the remaining 16 infants were grouped into the non-allergic group. According to the analysis, the level of 2′-FL, one of the most abundant HMOs, ingested by infants in the non-allergic group was higher than that of the allergic group at all five stages of lactation, with a significant difference occurring at the M3 period. Additionally, numerous studies have elucidated the vital importance of HMOs in shaping the healthy intestinal microenvironment of infants. Given these facts, the focus of this study was on the association between HMOs, infants’ and young children’s allergic diseases, and the gut microbiome. Over the next 5 years, we successfully conducted a long-term follow-up survey of the above 20 mother–infant pairs. Another eight pairs tracked from the MUAI study were incorporated into further studies on the long-term associations between early HMO intake and allergic diseases.

### 3.2. Allergen Testing and Regrouping in Young Children

After mothers signed the informed consent form for subjects, the children underwent standardized allergen testing at Tianjin Children’s Hospital using a panel of 34 inhalant and food allergens. Children with a sensitization score ≥ 1 to any allergen were classified as allergic. Detailed allergen sensitization scores for each child are provided in Supplementary Table S3. Based on these results, children were divided into allergic and non-allergic groups for subsequent analyses. Multiple children were mainly allergic to three allergens, including milk, Penicillium notatum/Cladosporium/Tobacco mold/Aspergillus Niger/Alternaria, and Dwarf ragweed/artemisia humulus/quinoa, to varying degrees. In view of the test results, the children were divided into allergic and non-allergic groups. Subsequently, we preliminarily explored the association between early HMO intake and allergic disease in early childhood, based on the more accurate grouping mentioned before. First, we found that a higher proportion of children in the non-allergic group than in the allergic group were nursed by Se+ mothers (76% vs. 58%). In addition, although the regrouped data did not show statistically significant differences, the median intake levels of the 17 detected HMOs and total HMOs during early life were consistently higher in the non-allergic group compared with the allergic group (Supplementary Table S4). These results indicated that higher levels of HMO intake in early life might be related to a lower incidence of allergic diseases in childhood.

### 3.3. Associations Between Early HMO Intake and Intestinal Microbiota Structure in Children

**Figure 2:**
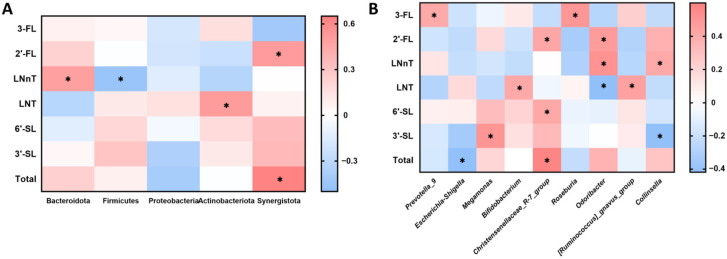
Effects of early human milk oligosaccharide (HMO) intake on intestinal microbiota structure in 5-year-old children. Correlation analyses between intestinal microbial abundance and six representative HMOs at (**A**) phylum level and (**B**) genus level. Correlations were assessed using Spearman correlation analysis. * *p* < 0.05. 3-FL, 3-Fucosyllactose; 2′-FL, 2′-Fucosyllactose; LNnT, Lacto-N-neo-tetraose; LNT, Lacto-N-tetraose; 6′-SL, 6′-Sialyllactose; 3′-SL, 3′-Sialyllactose.

To investigate how HMO intake during infancy influences the structural composition and functional encoding of the gut microbiota in early childhood, correlation analyses were conducted between intestinal microbial abundance and six predominant, structurally representative HMOs (2′-FL, 3-FL, LNT, LNnT, 3′-SL, and 6′-SL) in HBM at different levels. As shown in Figure 2A, intake of the six HMOs was significantly correlated with the abundance of four major phyla in the intestines of young children, including Bacteroidota, Firmicutes, Actinobacteriota, and Synergistota. Figure 2B shows 9 of the top 50 bacteria genera significantly associated with early HMO intake at the genus level. Therein, the total intake of six HMOs is significantly negatively correlated with the pathogenic bacteria *Escherichia-shigella*. Moreover, several distinct HMOs are found to be significantly positively associated with the abundance of several potentially beneficial bacterial genera, including *Megamonas*, *Christensenellaceae*_R-7_group, *Roseburia*, and *Collinsella*. These results suggest that differing levels of early HMO intake are associated with differences in the structural composition of intestinal microbiota in young children.

### 3.4. Association Between Allergic Diseases and Gut Microbiota Composition in Young Children

**Figure 3:**
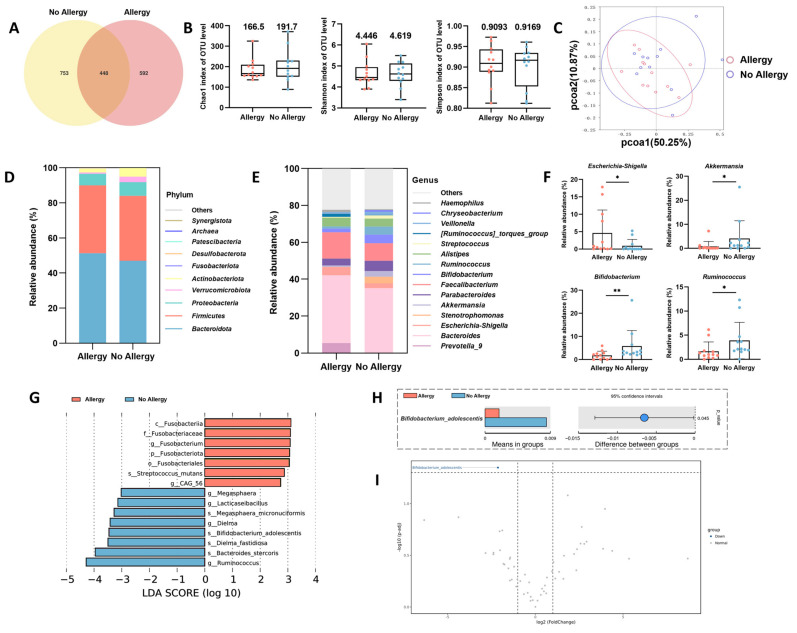
Differences in gut microbiota composition between allergic and non-allergic children at 5 years of age. (**A**) Venn diagrams of among groups. (**B**) Chao1 index, Shannon index, and Simpson index. (**C**) Principal coordinate analysis (PCoA). (**D**) Bacterial taxonomic composition at the phylum level. (**E**) Bacterial taxonomic composition at the genus level. (**F**) Relative abundance of the significantly differential flora at the genus level. (**G**) Branch diagram depicting the output of the linear discriminant analysis effect size (LEfSe) analysis. (**H**,**I**) Statistical comparisons of representative differential bacteria. Data are shown as the mean ± SD (*n* = 12). Group comparisons were performed using Kruskal–Wallis test or the Student’s *t*-test as appropriate. * *p* < 0.05, ** *p* <0.01.

To investigate potential differences in the gut microbiota structure between the group of allergic and non-allergic children, 16S rRNA sequencing was performed in the study to examine the fecal microbiota collected uniformly the day before allergen testing. Clustering all high-quality sequences with 97% identity, the Venn diagram showed that the allergic and non-allergic groups resulted in 1040 and 1201 operational taxonomic units (OTUs), respectively, including 448 shared and specific OTUs of the two microbial populations (Figure 3A). α-diversity (Chao1 index, Shannon index, and Simpson index) was used as a measure of species richness and diversity in the gut microbiota within communities. As shown in Figure 3B, the medians of these indices were lower in the allergic group than in the non-allergic group, albeit with no significant difference. Principal component analysis (PCoA) was used to analyze β-diversity as a measure of diversity across microbial communities, and the principal coordinates on the two-dimensional plane were 50.25% and 10.87%, with a slight shift in microbial structure between the allergic and non-allergic groups (Figure 3C).

To further clarify the differences in bacterial community structure between the two groups, the species richness at the phylum level (top 10) and the genus level (top 15) is shown in Figure 3D,E. By observing the results of the species richness histogram, it can be found that at the phylum level, the allergy group had a lower abundance of *Verrucomicrobiota* and *Actinobacteriota* and a higher abundance of *Fusobacteriota* and *Desulfobacterota* (Figure 3D). Bacterial genera with significant abundance differences between the two groups are listed in Figure 3F. Conditional pathogens such as *Escherichiae-shigella* were more abundant in the allergy group, whereas *Bifidobacterium*, *Akkermansia*, and *Ruminococcus*, which were positively correlated with infant health, were more abundant in the non-allergic group.

Differential gut microorganisms between the allergic and non-allergic groups were subsequently explored by linear discriminant analysis (LEfSe). As shown in Figure 3G, at the genus level, the level of the pathogenic bacterium *Fusobacterium* was significantly higher in the allergic group, while *Megamonas*, *Lacticaseibacillus*, and *Ruminococcus* were enriched in the non-allergic group. At the species level, the major caries-causing bacterium *Streptococcus mutans* and the probiotic *Bifidobacterium adolescentis* (*B. adolescentis*) were enriched in the allergic and non-allergic groups, respectively. To further clarify the microbial biomarker differences between the two groups, a *t*-test was performed and revealed notable differences in the abundance of *B. adolescentis* at a significance threshold of *p* < 0.05 or even *p* < 0.01 (Figure 3H,I).

### 3.5. Determination of Short-Chain Fatty Acid (SCFA) Content

**Figure 4:**
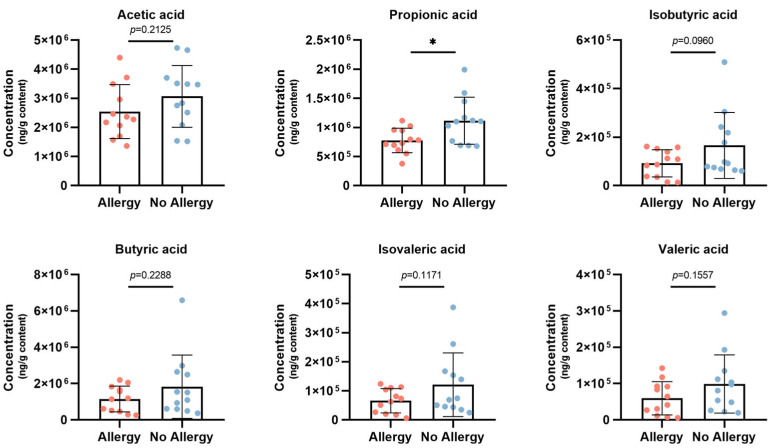
Short-chain fatty acid (SCFA) concentrations in allergic and non-allergic children at 5 years of age. Data are presented as mean ± SD. Group comparisons were conducted using Kruskal–Wallis one-way ANOVA. * *p* < 0.05.

SCFAs, metabolites produced via fermentation of insoluble substrates by beneficial bacteria in the intestinal microbiota, have been proven to improve the intestinal environment and regulate intestinal immune homeostasis by binding to G-protein-coupled receptors (GPRs), signaling molecules which may play a complementary role in the alleviation of allergic diseases [36]. Upon determining the levels of SCFAs in the intestines of subjects in both groups, all six SCFAs were higher in children in the non-allergic group compared to the allergic group. Among them, there was a significant difference in propionic acid content between the two groups, with a 1.44-fold increase in the non-allergic group in comparison to the allergic group (Figure 4).

### 3.6. Correlation Analysis Between Bifidobacterium spp. Abundance and HMO Intakes

**Figure 5:**
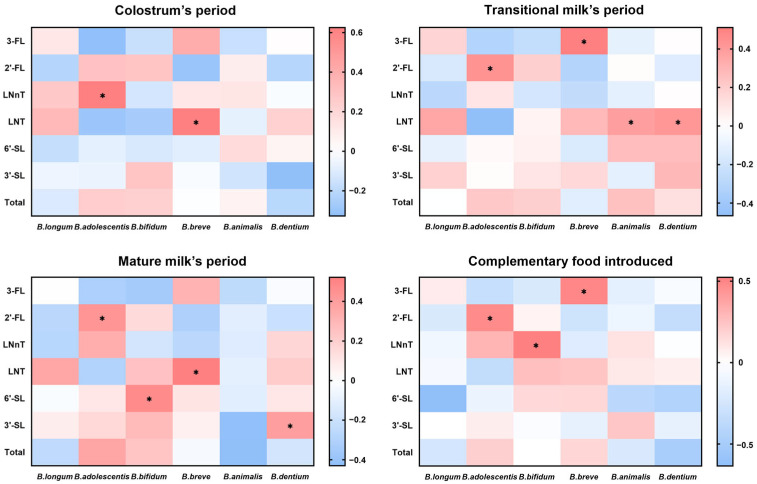
Correlation analysis between early-life HMO intake and the abundance of *Bifidobacterium* spp. in 5-year-old children. Correlations were assessed using Spearman correlation analysis. * *p* < 0.05. 3-FL, 3-Fucosyllactose; 2′-FL, 2′-Fucosyllactose; LNnT, Lacto-N-neo-tetraose; LNT, Lacto-N-tetraose; 6′-SL, 6′-Sialyllactose; 3′-SL, 3′-Sialyllactose.

To further corroborate the correlation between the abundance of *Bifidobacterium* in the intestinal microbiota and HMO intake in early life, six detected species of *Bifidobacterium* species, including *B. longum*, *B. adolescentis*, *B. bifidum*, *B. breve*, *B. animalis*, and *B. dentium*, were correlated with the levels of HMOs in different periods. Referring to the correlation heatmap in Figure 5, the differential microbial strain biomarker *B. adolescentis* demonstrated a significant correlation with a certain HMO at each of the four different lactation periods, especially with a significant positive correlation with the 2′-FL content in early intake. In addition, *B. breve* was also observed to be significantly positively related to 3-FL and LNT content. Taken together, our study suggests that early-life HMO intake is associated with allergic outcomes and gut microbiota composition in young children, potentially involving *B. adolescentis*.

## 4. Discussion

Infancy and early childhood represent critical windows for immune system development, during which tolerance to commensal microbes is established and resistance to pathogens and allergens is shaped [37]. The gut microbiota has been recognized as an important system for the organism’s healthy development, as it plays a regulatory role through interactions with the “axes” of various organs. The richness of intestinal microbiota in healthy infants gradually increases in early life. Also, it contributes to maintaining immune development and homeostasis later in life through the development of gut-associated lymphoid tissues (GALTs) [38,39]. Early HMO interventions have been shown to induce the predominant microbe *Bifidobacterium* and its metabolites to exhibit pertinent health-promoting properties [40,41]. In addition to their ability to regulate the infant gut microenvironment by exerting prebiotic benefits, HMOs also have strong immunomodulatory potential. Therefore, our study aimed to provide a stronger theoretical foundation for associations between HMOs and microbial–immune relationships.

In this observational study, a correlation between higher levels of 2′-FL intake and lower incidence of allergic symptoms during infancy, tracked from the M2 to M3 lactation period, was demonstrated. This finding is consistent with a report by Sprenger et al. stating that feeding breast milk with FUT2-dependent HMOs reduced the morbidity of allergic manifestations in these children at 2 years [42]. Furthermore, another finding reported that supplementation of infant formula with two HMOs, including 2′-FL, reduced the risk of cow’s milk sensitization and corrected gut microbiota dysbiosis [31]. These findings contribute to a rationale about the impact of early HMO intake on the development of allergic diseases and the encoding of microbial profiles in young children. Through five years of continuous follow-up using standardized hospital-based allergen testing, allergic manifestations in the young children of our study participants were assessed. According to the allergy vs. non-allergy regrouping, we analyzed the correlation between the level of breast milk HMO intake in infancy and the incidence of allergic diseases in young children and discovered that the median intake of 17 HMOs and the total amount of HMOs was higher in the non-allergic group of children than in the allergic group.

Since the shaping of intestinal microbiota composition in early life has a decisive and sustained impact on gut microecological development thereafter, we explored the direct association between HMO intake during infancy and gut homeostasis in young children. Our study found that six abundant HMOs were significantly negatively associated with *Escherichia-shigella*, a pathogen reported to induce acute toxicity, diarrhea, and fever in young children, suggesting that breastfeeding or HMO supplementation may be associated with a lower abundance of potential pathogens. In addition, we reported several genera of beneficial bacteria that were significantly and positively related to HMO intake levels. For instance, *Christensenellaceae*_R-7_group abundance was positively correlated with the proteolytic metabolism of animal proteins and intestinal metabolites, promoting nutrient absorption and digestion [43]. The key gut microbial genus *Roseburia* is recognized for its substantial production of butyric acid and has been associated with beneficial effects in conditions such as irritable bowel syndrome, obesity, and allergic diseases [44,45]. *Ruminococcus*, enriched in the intestine of children in the non-allergic group, aligned with the finding reported by Tun et al. that infants’ exposure to pets increased *Ruminococcaceae* abundance, which was negatively associated with childhood allergy [46].

HMOs have previously been demonstrated to modulate the gut microbiota and enhance the production of beneficial metabolites, particularly SCFAs, which are widely recognized as signaling molecules contributing to intestinal homeostasis in the healthy development of infants and young children [47,48]. Our study also revealed that the more favorable gut microbiota composition observed in the non-allergic group was associated with improved outcomes and elevated levels of SCFAs. Among them, propionic acid, which was significantly different between the two groups, has been shown to have efficacy in relieving allergies. For instance, Trompette et al. conducted an intervention study in mice using propionic acid and observed an enhancement in the production of macrophage and dendritic cell precursors. Additionally, the impaired Th2 immune response was restored in the treated mice [49]. Beyond microbial and metabolic pathways, epigenetic mechanisms may also play a critical role in mediating the long-term effects of early-life HMO exposure on immune development and allergy risk [50]. Evidence suggests that HMOs and their derived microbial metabolites, SCFAs, can inhibit histone deacetylases (HDACs) and increase histone acetylation levels [51]. Through these epigenetic modifications, SCFAs may regulate intestinal epithelial differentiation and immune cell function, thereby contributing to immune tolerance and influencing the development of allergic diseases [49]. In addition to HMOs, emerging evidence indicates that plant polyphenols, secondarily present in human milk, can interact with HMOs to jointly modulate infant gut microbiota maturation and metabolic profiles, thereby influencing immune development and early-life allergy susceptibility [52]. As maternal dietary polyphenols were not explicitly incorporated into the present analysis, potential synergistic effects between HMOs and other milk-derived bioactive compounds cannot be fully excluded. Nevertheless, these preclinical results further confirmed that a stable intestinal microecology contributed to the restoration of dysfunctions in the body’s immune system.

Interactions between the rich mucosal-associated lymphoid tissue and trillions of intestinal microorganisms in early childhood are vital for the generation of immune tolerance, and different microbiota compositions can induce distinguishing patterns of immune responses. Notably, *B. adolescentis* was revealed in this study as a differential genus in the gut of allergic and non-allergic children. The health benefits of this commensal are gradually gaining widespread attention in recent research. For example, a study found that fecal *B. adolescentis* levels were lower in patients with IBD than in normal subjects by comparing cohort studies and GMrepo databases, and *B. adolescentis* could ameliorate DSS-induced chronic colitis by stimulating protective Treg/Th2 responses and gut microbiota remodeling [53]. In addition, a cross-sectional study of the composition and function of the gut microbiota in Sardinian centenarians reported that *B. adolescentis*, which typically decreases with age, was relatively enriched in centenarians [54]. Moreover, Shujie Chen et al. elucidated that *B. adolescentis* improved the healthy lifespan of several species, including prematurely aging mice, *Drosophila melanogaster*, and *Heterorhabditis elegans*, through the ability to regulate catalase activity and host metabolism [55]. Consistent with our findings, numerous studies have evidenced the positive benefits of *B. adolescentis* in a “younger, healthier” intestinal environment. Through correlation analysis, we found a positive association between higher levels of 2′-FL intake in early life and a higher abundance of *B. adolescentis* in the intestinal tract of non-allergic young children, which emphasizes the potential mediating role of the HMO-degrading bacterium *B. adolescentis* in the association between early-life HMO intake and allergic outcomes, supporting its relevance as a candidate microbial biomarker.

Several limitations of this study should be acknowledged. Although additional participants from the same MUAI cohort were recruited to compensate for attrition, the final sample size for long-term follow-up (*n* = 20) remained modest, which may reduce statistical power and introduce potential selection bias despite identical eligibility criteria across retained, lost, and supplemented participants. The geographically homogeneous Tianjin-based population strengthened internal validity but restricted generalizability to other ethnic, regional, or socioeconomic contexts, warranting validation in multi-center and multi-ethnic populations. Maternal genetic determinants of HMO biosynthesis, including FUT2 and FUT3 genotypes or secretor status, were not directly assessed, restricting mechanistic interpretation despite quantitative measurement of HMO exposure across five lactation stages. Reliance on parental questionnaires for infant allergy assessment and single-time-point sampling per lactation stage may have introduced measurement error, although objective standardized allergen testing was incorporated at the 5-year follow-up. In addition, although maternal characteristics, feeding practices, and environmental exposures were collected and key confounders were adjusted for, residual confounding may persist given the observational design and long follow-up duration. Collectively, these constraints limit causal inference and underscore the need for future studies incorporating larger and more diverse cohorts, maternal genotyping, detailed exposure assessment, and advanced statistical or randomized designs to establish definitive relationships.

## 5. Conclusions

Our previous finding that varying levels of HMO intake are associated with significant effects on the development of allergic symptoms in infants led to hypotheses on whether the intake of HMOs in early life has a longer-term impact on allergic disease development and gut microbiological architecture in young children. Hence, we conducted a 5-year long-term follow-up of mothers concerned about their children’s allergic conditions in the MUAI study. Through our current analysis, we found that higher levels of HMO intake during infancy were associated with a greater enrichment of *B. adolescentis* in non-allergic children and with more favorable health-related profiles in early childhood. Nevertheless, given the observational design, modest sample size, and potential residual confounding, these findings should be interpreted as associative rather than causal and warrant confirmation in larger and more diverse cohorts. This study supports the value of HMO supplementation and provides advanced strategies to potentially reduce the prevalence of allergic diseases in infants and young children to improve long-term health outcomes.

## Data Availability

All data supporting this study’s findings are included in the article and Supplementary Information. Further inquiries can be directed to the corresponding author.

## Supplementary Materials

The following supporting information can be downloaded at https://www.mdpi.com/article/10.3390/nu18040624/s1, Table S1: Baseline characteristics of mother–infant pairs by follow-up status; Table S2: Intake levels of 17 human milk oligosaccharides (HMOs) across five lactation stages in allergic and non-allergic infants; Table S3: Results of standardized allergen sensitization testing in the study children; Table S4: Comparison of early-life intake of 17 and total HMOs between allergic and non-allergic children.

## Abbreviations

The following abbreviations are used in this manuscript:

HMO	Human milk oligosaccharide
HBM	Human breast milk
2′-FL	2′-Fucosyllactose
DFL	Difucosyllactose
LNFP I	Lactose-*N*-fucosidase
3′-SL	3′-Sialyllactose
LNT	Lacto-N-tetraose
LNnT	Lacto-N-neo-tetraose
FA	Food allergy
AAS	Allergic asthma
AD	Atopic dermatitis
CMPA	Cow’s milk protein allergy
CECAQ	Comprehensive Early Childhood Allergy Questionnaire
OTU	Operational taxonomic unit
SCFA	Short-chain fatty acid
GPR	G-protein-coupled receptor
